# CCL2/CCR2 Axis Promotes the Progression of Salivary Adenoid Cystic Carcinoma via Recruiting and Reprogramming the Tumor-Associated Macrophages

**DOI:** 10.3389/fonc.2019.00231

**Published:** 2019-04-09

**Authors:** Zihui Yang, Huan Li, Weiqi Wang, Jianying Zhang, Sen Jia, Jun Wang, Jianhua Wei, Delin Lei, Kaijin Hu, Xinjie Yang

**Affiliations:** ^1^State Key Laboratory of Military Stomatology & National Clinical Research Center for Oral Diseases & Shaanxi Clinical Research Center for Oral Diseases, Department of Oral and Maxillofacial Surgery, School of Stomatology, The Fourth Military Medical University, Xi'an, China; ^2^Department of Oral and Maxillofacial Surgery, Xi'an Medical University, Xi'an, China

**Keywords:** salivary adenoid cystic carcinoma (SACC), tumor-associated macrophages (TAMs), CCL2, CCR2, tumor progression

## Abstract

**Objective:** The present study investigated the roles and underlying mechanism of CCL2/CCR2 axis in the interactions between tumor cells and tumor-associated macrophages (TAMs) during the progression of salivary adenoid cystic carcinoma (SACC).

**Methods:** Immunohistochemical staining and survival analysis were performed to study the correlation and clinical value of CD68, CD163, CCL2, and CCR2 expression in SACC cases. CCL2 silencing by RNA interference and CCR2 blocking by CCR2 specific antagonist (RS504393) were performed. ELISA, qRT-PCR, western blot, immunofluorescence, flow cytometry, CCK8, scratch wound healing, and transwell assays were used to explore the functional roles and possible mechanism of CCL2/CCR2 axis in the interactions between SACC cells and TAMs. The effects of targeting TAMs by blocking the CCL2/CCR2 axis were investigated in a xenograft mice model with SACC cells.

**Results:** The high infiltration of TAMs marked by CD68 and high infiltration of M2 TAMs marked by CD163 were significantly correlated with the expression of CCL2 and CCR2 in SACC tissues. Notably, the high infiltration of TAMs and the overexpression of CCL2 were obviously associated with the clinical progression and poor prognosis of SACC. SACC cells derived CCL2 could activate its receptor CCR2 expression in TAMs *in vitro*. The *in vitro* results further indicated that the SACC cells derived CCL2 was involved in the recruitment, M2 polarization, and GDNF expression of TAMs through the CCL2/CCR2 axis. Meanwhile, TAMs derived GDNF promoted the proliferation, migration, and invasion of SACC cells through the GDNF/p-RET pathway. Treating immunodeficient mice with the CCR2 antagonist (RS504393) greatly inhibited the infiltration of TAMs and the tumorigenicity of SACC cells.

**Conclusion:** These new findings indicated that the CCL2/CCR2 axis promoted the progression of SACC cells via recruiting and reprogramming TAMs. Targeting TAMs by blocking the CCL2/CCR2 axis might be a prospective strategy for SACC therapy.

## Introduction

Salivary adenoid cystic carcinoma (SACC) is one of the most commonly diagnosed salivary gland malignancies, accounting for about 10% of all salivary gland neoplasms (1, 2). The typical characteristics of SACC include the recurrent growth, aggressive invasion, hematogenous metastasis, and chemotherapy refractory (2–4). Despite the great efforts paid in the treatment of SACC, the long-term prognosis of patients suffering SACC is still pessimistic (4, 5). Therefore, a better understanding of the molecular mechanism is needed to illuminate the aggressive behavior and guide the development of novel therapies for SACC.

Tumor microenvironment consists of complex cellular ecology that establishes the potential of the neoplasm (6). Substantial evidences demonstrated that the macrophages could be recruited and activated by the tumor cells (7–9). Macrophages that infiltrate into the malignant tumors are referred to as the tumor-associated macrophages (TAMs), which closely related to the progression and the prognosis of the malignancies (9, 10). TAMs were found to accelarate the growth, invasion, and metastasis of multiple malignancies, including lung cancer, breast cancer, and the hepatocellular carcinoma (7, 10, 11). Targeting TAMs was considered to be a promising therapeutic strategy for malignant tumors (10, 12). However, the role of TAMs in the progression of SACC is not elucidated so far. Thus, studying the reciprocal interactions between SACC and TAMs might provide new perspectives for the mechanism of SACC development.

Chemokines are important modulators that drive tumor development (13). CCL2, also named monocyte chemotactic protein-1, is a potent chemokine for the recruitment of TAMs (14). Accumulated studies indicated that CCL2 promoted the recruitment of TAMs via activating its receptor CCR2 in the process of tumor invasion and metastasis (14, 15). Recent studies demonstrated that the CCL2/CCR2 axis is responsible for the M2 polarization of TAMs, thereby shaping a tumor-supportive environment (11, 16, 17). Growing preclinical studies showed that targeting TAMs by blocking the CCL2/CCR2 axis inhibited the tumor development and might be a potential therapeutic revenue for several malignancies (11, 17, 18). However, little is known about the role and molecular mechanism of the CCL2/CCR2 axis in the progression of SACC.

In the present study, we explored the clinical value of the CD68 (TAMs marker), CCL2, and CCR2 expression in SACC tissues. We further investigated the roles of the CCL2/CCR2 axis in the interactions between SACC cells and TAMs, and explored the underlying molecular mechanism. Moreover, we investigated whether blockade of the CCL2/CCR2 axis by CCR2 specific antagonist (RS504393) could inhibit the tumorigenicity of SACC cells in the xenograft tumor model. This study is supposed to better delineate the interactions between SACC cells and TAMs, and provide an innovative therapeutic strategy for SACC via blocking the CCL2/CCR2 axis.

## Materials and Methods

### Patients and Samples

This study was approved by the Medical Research Ethics Committee of The Fourth Military Medical University. The informed consents were obtained from all the patients. Paraffin-embedded specimens of 71 primary SACC and 50 normal salivary gland tissues were collected from our affiliated hospital archives between 2007 and 2012. The fresh SACC tissues were obtained from 5 SACC patients. All SACC patients were followed up by medical records or telephoning.

### Immunohistochemical Staining

The paraffin-embedded samples were cut into 4 μm sections for use. The immunostaining procedures were performed as described in our previous studies (19, 20). Briefly, after incubation with the primary antibodies including CD68 (1:200, Santa Cruz, USA), CD163 (1:200, Abcam, USA), CCL2 (1:400, Abcam, USA), CCR2 (1:400, Abcam, USA), F4/80 (1:400, Abcam, USA), and Ki-67 (1:400, Abcam, USA) at 4°C overnight, the specimens were processed using secondary antibody kits (SPlink Detection Kits, China) and stained with 3'-diaminobenzidine and hematoxylin.

All the stained sections were blindly assessed by two independent experienced pathologists. For CD68, CD163, and F4/80 evaluation, the numbers of positive cells were counted in 5 random fields (400x magnification) and obtained the mean number. The number of CD68^+^ cells <25 per field was considered as low expression, and the number ≥25 per field was considered as high expression. The number of CD163 cells <18 per field was considered as low expression, and the number ≥18 per field was considered as high expression. For CCL2 and CCR2 evaluation, the intensity of staining (weak = 1; intensive = 2) and the percentage of positive cells (<25% = 1; 25 to 50% = 2; 50 to 75% = 3; >75% = 4) were assessed in 5 random fields (400x magnification). The scores of staining intensity and percentage of positive cells were multiplied to generate a final score. Then, the tumor specimens were divided into the low expression (score 1, 2, and 3) and the high expression (score 4, 6, and 8) according to the score. For Ki-67 evaluation, the Ki-67 index was examined by calculating the percentage of Ki-67 positive cells per 1,000 cells under the 400x magnification.

### Transmission Electron Microscopy

The fresh SACC tissues were fixed with 4% glutaraldehyde, cut into 1 mm^3^ blocks, and fixed with 4% glutaraldehyde at 4°C overnight. Subsequently, the blocks were sectioned into 70 nm thickness and stained with uranyl acetate and lead citrate. The material was observed under transmission electron microscopy (FEI, TECNAI G2, USA).

### Cell Culture

The human adenoid cystic carcinoma cell line SACC-83 and SACC-LM (21) were obtained from Peking University School of Stomatology, and the Human acute monocytic leukemia cell line THP-1 was obtained from ATCC. SACC-83, SACC-LM, and THP-1 cells were cultured in RPMI-1640 supplemented with 10% fetal bovine serum, 100 U/mL penicillin, and 100 mg/mL streptomycin in a humidified atmosphere of 95% air and 5% CO_2_ at 37°C. For the generation of macrophages, THP-1 cells were induced by phorbol 12-myristate 13-acetate (100 nM, Sigma, USA) for 24 h.

CCR2 inhibition in macrophages was performed by incubation with RS504393 (50 ng/mL, Tocris, Bristol, UK). RET inhibition in SACC-83 cells was performed by incubation with pyrazolo-pyrimidine-1 (PYP1, 5 μM, R&D systems, USA).

### RNA Interference and Cell Co-culture

The sequences of shRNAs were listed in Table S1, including a non-specific sequence (shNC) and two different sequences targeting human CCL2 (shRNA1 and shRNA2). shRNAs were cloned into the lentiviral vector pLKO.1-TRC-GFP (Addgene) and then packaged and transfected into 293T cells (ATCC, USA) using Lipofectamine™ 2000 Transfection reagent (Invitrogen, USA). After 48 h of transfection, the lentivirus was collected, filtered, and delivered into SACC-83 cells in the presence of polybrene (6 μg/mL). Fouty-eight hours later, the transduced SACC-83 cells were treated with puromycin (1 mg/mL) to select resistant clones. The resultant stable clones were designated as shNC, sh1, and sh2 respectively.

For the co-culture experiment, macrophages were seeded onto the 6-well plates at the density of 3 × 10^6^ cells per well. SACC-83, shNC, or sh2 cells (3 × 10^6^ per well) were seeded onto 0.4-μm pore membrane inserts (Corning, NY, USA). Cells were co-cultured in RPMI-1640 supplemented with 0.1% bovine serum albumin (BSA) for 48 h. The above procedures for SACC-83 cells were also performed on SACC-LM cells.

### Preparation of Conditioned Media

Procedures to generate conditioned media were according to previous studies (11, 22, 23). For the preparation of the macrophages conditioned media, macrophages were cultured with serum-free RPMI-1640 supplemented with 0.1% BSA for 48 h. For the preparation of the SACC-83 conditioned media, SACC-83 cells were incubated with serum-free RPMI-1640 supplemented with 0.1% BSA for 48 h. For the preparation of the TAMs conditioned media, macrophages were incubated with the SACC-83 conditioned media for 48 h.

Neutralization of GDNF in the conditioned media was performed using GDNF neutralizing antibody (GDNF NAb, 2 μg/mL, GeneTex, USA) for 2 h before the experiment.

### Immunofluorescence Assay

Cells were seeded onto the 24-well plates (Corning, USA) at the density of 5 × 10^4^ per well for 24 h. Then the cells were fixed with 4% paraformaldehyde for 15 min and permeabilized with 0.1% Triton X-100 for 25 min. Subsequently, the cells were incubated with the CCL2 primary antibody (1:200, Affinity, USA) overnight at 4°C followed by the incubation with Cy3-conjugated anti-rabbit IgG (1:800, Abbkine, USA) for 1 h at room temperature. DAPI (Fanbo, China) was used for nuclear staining.

### Enzyme-Linked Immunosorbent Assay (ELISA)

The concentrations of CCL2 and GDNF in the conditioned media were measured by ELISA kits (R&D Systems, USA) following the manufacturer's instructions. Briefly, 100 μL samples were loaded onto the 96-well plates and incubated at room temperature for 2 h. Then the plates were incubated with the primary antibody for another 2 h at room temperature. After adding the substrate solution, the immunoreactivity was evaluated at 450 nm by the Vmax Kinetic microplate reader (Sunnyvale, USA).

### Flow Cytometry Assay

A number of 1 × 10^6^ cells were incubated with PE-CD163 (1:20, Invitrogen, USA) or FITC-CD206 (1:20, Invitrogen, USA) antibodies according to the manufacturer's instructions. Flow cytometry was performed by Cytomics™ FC 500 (Beckman Coulter, USA).

### CCK8 Proliferation Assay

Cells were seeded at the density of 2 × 10^3^ cells per well onto the 96-well plates and then incubated with the conditioned media for 12, 24, 36, and 48 h respectively. The cell proliferation was evaluated using a CCK8 kit (7 Sea biotech, China) according to the manufacturer's instructions.

### Scratch Wound Healing Assay

Cells were seeded onto the 6-well plates. When reaching 90% of confluency, the cells were wounded across the center of each well by a sterile pipette tip. After 24 h of incubation in the indicated conditioned media, images of the cells were photographed by an inverted microscope (Olympus, Center Valley, PA).

### Migration and Invasion Assays

A number of 1 × 10^5^ tumor cells or macrophages in 200 μL serum-free RPMI-1640 were seeded onto the transwell inserts (for migration assay, 8 μm, Corning, USA) or Matrigel-coated inserts (for invasion assay, 8 μm, Corning, USA) of the 24-well plates. The lower chamber was filled with 600 μL conditioned media as the chemoattractant. After 12 h of incubation, the cells on the upper chamber were carefully removed. The cells that passed the membrane were fixed with 4% formaldehyde and stained with 1% crystal violet. The evaluation of migration and invasion were performed by counting the number of stained cells in five random fields (400x magnification).

### Quantitative Real-Time Polymerase Chain Reaction (qRT-PCR)

Total RNA was extracted from cells using the RNA Extraction Kit (Takara Bio Inc. Otsu, Japan) according to the manufacturer's instructions. The RNA samples were reversed to cDNA using the Prime Script RT Master Mix (Takara). The qRT-PCR was performed using the SYBR Premix Ex Taq Kit (Takara). The primer sequences of CCR2, TNF-α, IL-1β, Arg1, IL-10, GDNF, and β-actin were listed in Table S2. β-actin mRNA served as the internal control.

### Western Blot

Total cell protein was separated on 10% SDS-PAGE (Pall Corporation, USA), and transfected to the membranes (EMD Millipore, USA). Then, the membranes were blocked with 5% BSA for 1 h and immunoblotted with anti-CCL2 (1:500, affinity, USA), anti-CCR2 (1:500, affinity, USA), anti-GDNF (1:500, Abcam, USA), anti-p-RET (1:500, affinity, USA), anti-RET (1:500, affinity, USA), or anti-β-actin (1:1,000, affinity, USA). The results were visualized with a chemiluminescence reagent (Thermo, USA).

### *In vivo* Tumorigenicity Assay

All of the procedures involving animals were approved by the Committee on Use of Live Animals for Teaching and Research of the Fourth Military Medical University. The male nude BALB/c mice, aged 6–8 weeks, were purchased from Shanghai SLAC Laboratory Animal Co. Ltd. SACC-83 cells (5 × 10^6^ per mouse) suspended in 0.2 mL PBS were inoculated subcutaneously into the back of each mouse (*n* = 6). One week after the inoculation, the mice were administrated orally with RS504393 (30 mg/kg, Tocris, Bristol, UK) dissolved in 100 μL of methylcellulose in the experiment group or with methylcellulose in the control group every 3rd day for 3 weeks. Tumor volumes were monitored weekly using calipers and calculated according to the formula: length × width^2^ × 0.5. Tumors were harvested at the end of 4 weeks. Then the tumor tissues were carefully separated, weighed and subjected to immunohistochemistry analysis for F4/80, CD163, and Ki67.

### Statistical Analysis

All the statistical analysis were performed using the SPSS 24.0 software (IBM, Armonk, NY, USA). The expression evaluation in the SACC and normal salivary gland tissues were performed by the *Fisher's exact test*. Correlations were assessed by the *Spearman rank correlation coefficient test*. Survival curves were evaluated by the *Kaplan–Meier analysis*, and the statistical significance was analyzed by the *log-rank test*. All the *in vitro* experiments were performed in triplicate. The *in vitro* and *in vivo* data were expressed as Mean ± SD and the statistical comparisons were performed by the *one-way ANOVA test* or *Students' t*-*test*. The *P* value < 0.05 was considered as statistically significant.

## Results

### Expression and Correlation of CD68, CD163, CCL2, and CCR2 in SACC

**Figure 1:**
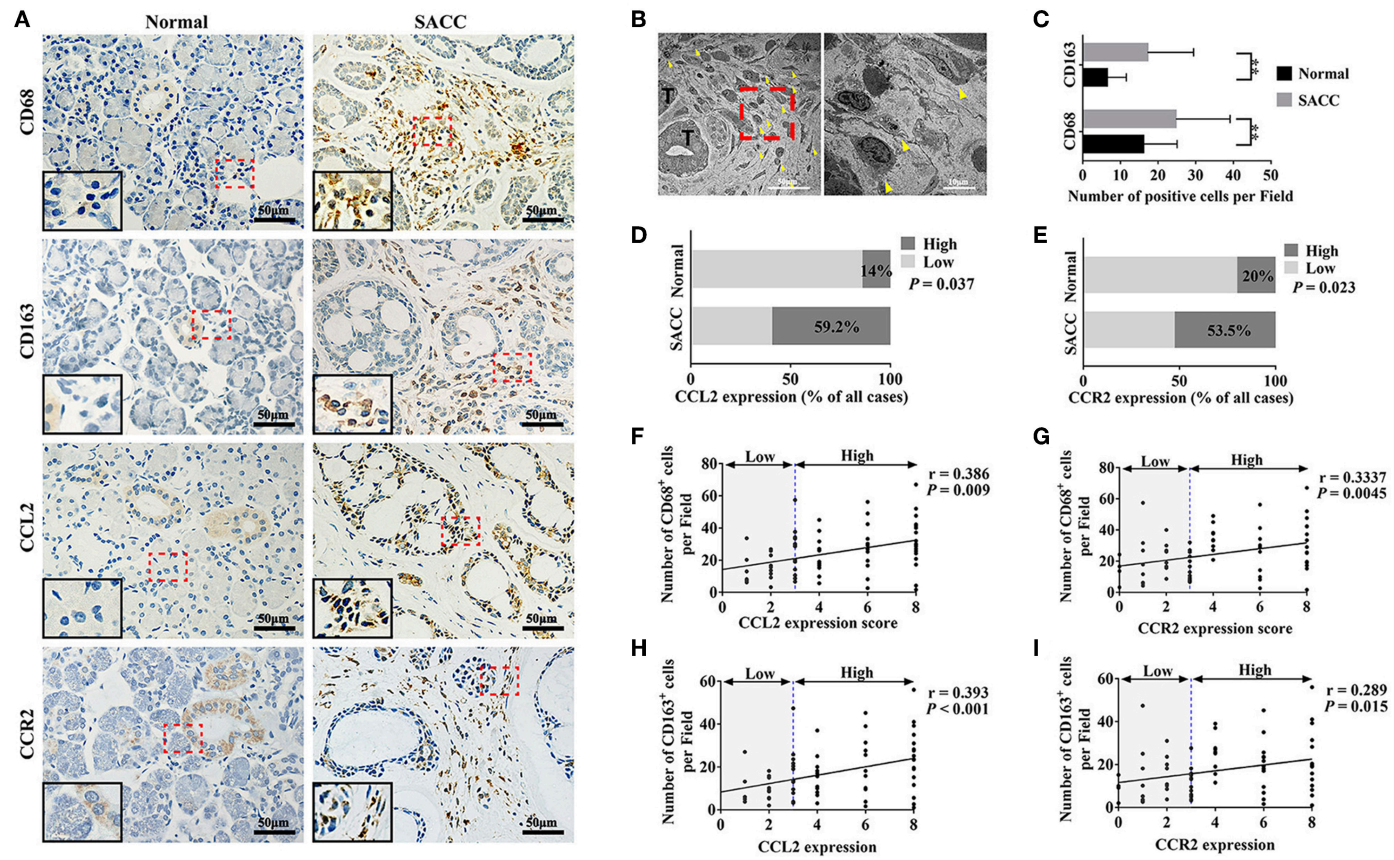
CCL2 and CCR2 were associated with the infiltration of CD68 marked TAMs and CD163 marked M2 TAMs in SACC tissues. **(A)** Representative immunohistochemical staining of normal salivary gland tissues and SACC tissues for CD68, CD163, CCL2, and CCR2. Bar = 50 μm. The magnification of the red dotted area are presented at the bottom of the left corner. **(B)** Ultrastructural view of TAMs in SACC tissues. The right image (bar = 10 μm) showed TAMs at high magnification of the red dotted area in the left image (bar = 50 μm). T represented the tumor cells. The yellow arrowheads showed the TAMs. **(C–E)** Analysis of CD68, CD163, CCL2, and CCR2 expression in the normal tissues and SACC tissues. **(F,G)** Analysis of the correlation between the expression of CCL2 and CCR2 with the number of CD68 positive (CD68^+^) cells in SACC tissues. **(H,I)** Analysis of the correlation between the expression of CCL2 and CCR2 with the number of CD163 positive (CD163^+^) cells in SACC tissues.^**^P < 0.01.

As shown in Figure 1A, CD68 and CD163 were mainly present in the membrane and cytoplasm of the stromal cells. The transmission electron microscopy results showed abundant infiltration of TAMs in SACC tissues (Figure 1B). The number of both CD68 and CD163 positive cells were significantly higher in SACC tissues than that in normal tissues (Figure 1C) (*P* < 0.01).

As shown in Figure 1A, CCL2 was mainly present in the cytoplasm of the tumor cells in SACC tissues. Whereas, in normal tissues, CCL2 was detected in some tuber cells and acinar cells. The expression of CCL2 was significantly higher in SACC tissues compared with that in normal tissues (Figure 1D) (*P* < 0.05). CCR2 was found in the cytoplasm of tumor cells and stromal cells in SACC tissues (Figure 1A). Whereas, in normal tissues, CCR2 was mainly present in some tuber cells. The expression of CCR2 was significantly higher in SACC tissues than that in normal tissues (Figure 1E) (*P* < 0.05). The expression of both CCL2 and CCR2 were significantly correlated with the number of CD68 marked TAMs (Figures 1F,G) and the number of CD163 marked M2 TAMs (Figures 1H,I) in SACC tissues (*P* < 0.01).

### Correlation Between the Expression of CD68, CD163, CCL2, CCR2, and the Clinicopathological Features of SACC

As shown in Table 1, the expression of CD68 was significantly associated with the clinical stage, perineural invasion, and distant metastasis of SACC (*P* < 0.05), whereas not with age, gender, tumor site, and tumor histotype (*P* > 0.05). The expression of CD163 was significantly associated with the clinical stage, perineural invasion, and distant metastasis of SACC (*P* < 0.05), whereas not with age, gender, tumor site, and tumor histotype (*P* > 0.05). The expression of CCL2 was significantly associated with the clinical stage, perineural invasion, and distant metastasis (*P* < 0.05), whereas not with age, gender, tumor site, and tumor histotype (*P* > 0.05). And the expression of CCR2 was significantly associated with the clinical stage and perineural invasion (*P* < 0.05), whereas not with age, gender, tumor site, tumor histotype, and distant metastasis of SACC (*P* > 0.05).

**Table 1:** Association of CD68, CD163, CCL2, CCR2 expression with clinicopathological parameters of SACC patients.

**Variables**	***n***	**CD68 expression**		***P*-value**	**CD163 expression**		***P*-value**	**CCL2 expression**		***P*-value**	**CCR2 expression**		***P*-value**
		**low**	**high**		**low**	**high**		**low**	**high**		**low**	**high**	
**GENDER**													
Male	36	16	20	0.410	17	19	0.284	15	21	0.887	18	18	0.549
Female	35	19	16		21	14		14	21		15	20	
**AGE**													
>50	41	19	22	0.563	21	20	0.652	17	24	0.902	17	24	0.325
≤50	30	16	14		17	13		12	18		16	14	
**SITE**													
Majaor	29	12	17	0.271	13	16	0.226	11	18	0.680	15	14	0.465
Minor	42	23	19		25	17		18	24		18	24	
**HISTOTYPE**													
C/T	53	28	25	0.310	28	25	0.842	24	29	0.195	26	27	0.458
S	18	7	11		10	8		5	13		7	11	
**STAGE**													
I+II	41	27	14	0.001^*^	27	14	0.016^*^	22	19	0.011^*^	24	17	0.018^*^
III+IV	30	8	22		11	19		7	23		9	21	
**PERINEURAL INVASION**													
Negative	46	27	19	0.033^*^	30	16	0.008^*^	23	23	0.035^*^	26	20	0.022^*^
Positive	25	8	17		8	17		6	19		7	18	
**METASTASIS**													
Negative	49	29	20	0.014^*^	32	17	0.003^*^	24	25	0.039^*^	26	23	0.099
Positive	22	6	16		6	16		5	17		7	15	

*T, tubular type; C, cribriform type; S, solid type*.

* *P < 0.05 by Spearman correlation test*.

### Expression of CD68, CD163, and CCL2 Were Correlated With the Prognosis of SACC Patients

**Figure 2:**
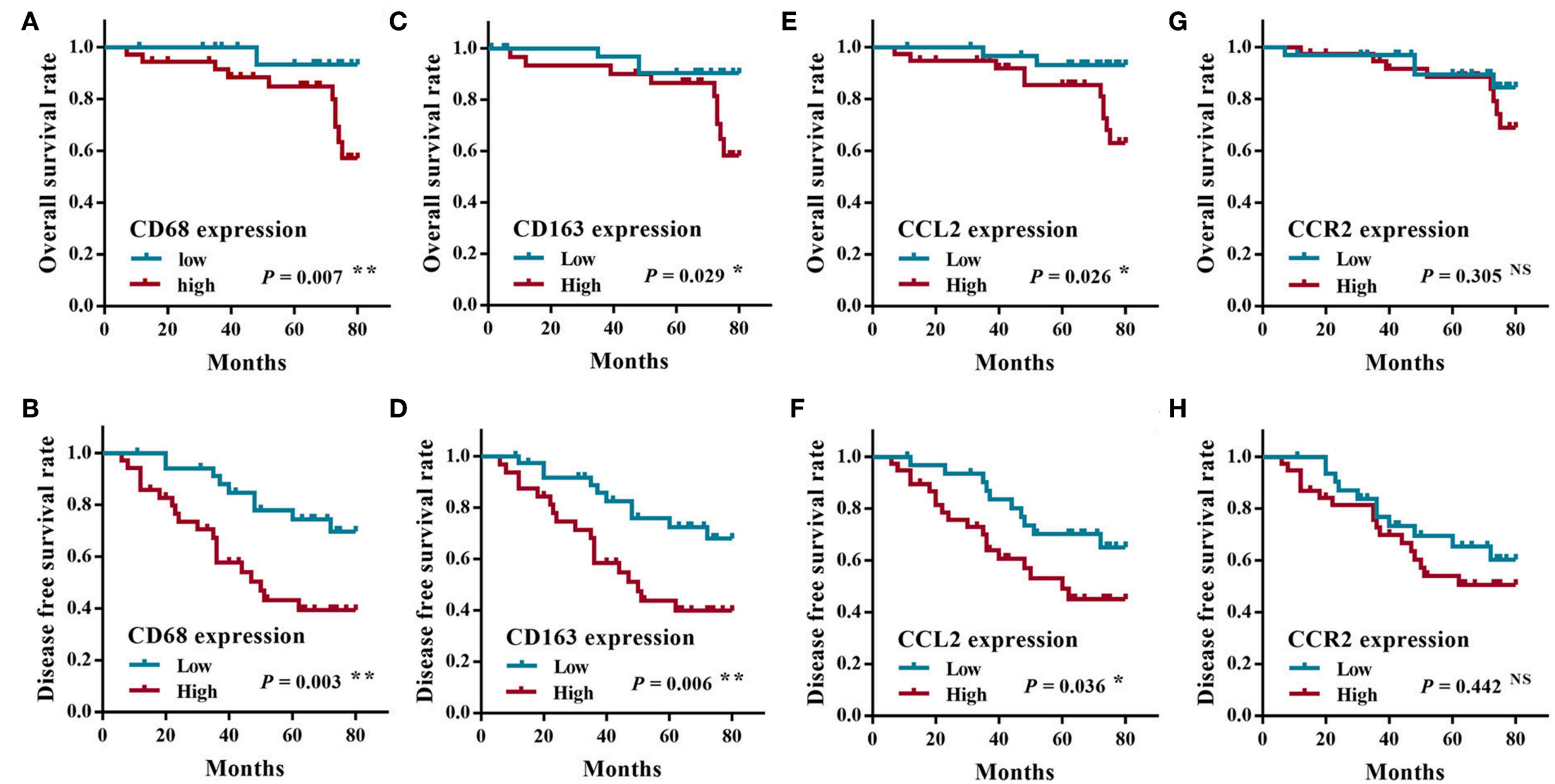
The survival analysis of SACC patients according to the expression of CD68, CCL2, and CCR2. The overall survival rate **(A)** and the disease free survival rate **(B)** of SACC patients with the high CD68 expression were significantly worse than those with the low CD68 expression (*P* < 0.01). The overall survival rate **(C)** and the disease free survival rate **(D)** of SACC patients with the high CD163 expression were significantly worse than those with the low CD163 expression (*P* < 0.05). The overall survival rate **(E)** and the disease free survival rate **(F)** of SACC patients with the high CCL2 expression were significantly worse than those with the low CCL2 expression (*P* < 0.05). The overall survival rate **(G)** and the disease free survival rate **(H)** of SACC patients had no significant correlation with the expression of CCR2 (*P* > 0.05). ^*^P < 0.05, ^**^P < 0.01, NS, no significance.

All patients were followed up until death or more than 5 years. The average period of follow-up was 68.45 ± 25.78 months (Mean ± SD; range, 7 to 140 months). At the end of this study, 12 patients (16.9%, 12/71) were lost during the follow-up, 16 patients (27.1%, 16/59) died of SACC, and 43 patients (72.9%, 43/59) were alive. Twenty-two patients (37.3%, 22/59) underwent distant metastasis of SACC (with 12 cases to lungs, 4 cases to the brain, 5 cases to lungs and bone, and 1 case to lungs and brain). The overall survival rate and the disease free survival rate of SACC patients were calculated according to the expression of CD68, CD163, CCL2, and CCR2. As shown in Figure 2, the high expression of CD68, CD163, or CCL2 were significantly correlated with the poor prognosis of SACC patients (*P* < 0.05), whereas the expression of the CCR2 was not associated with the prognosis of SACC patients (*P* > 0.05).

### SACC Cells Derived CCL2 Activated the CCR2 Expression in TAMs

**Figure 3:**
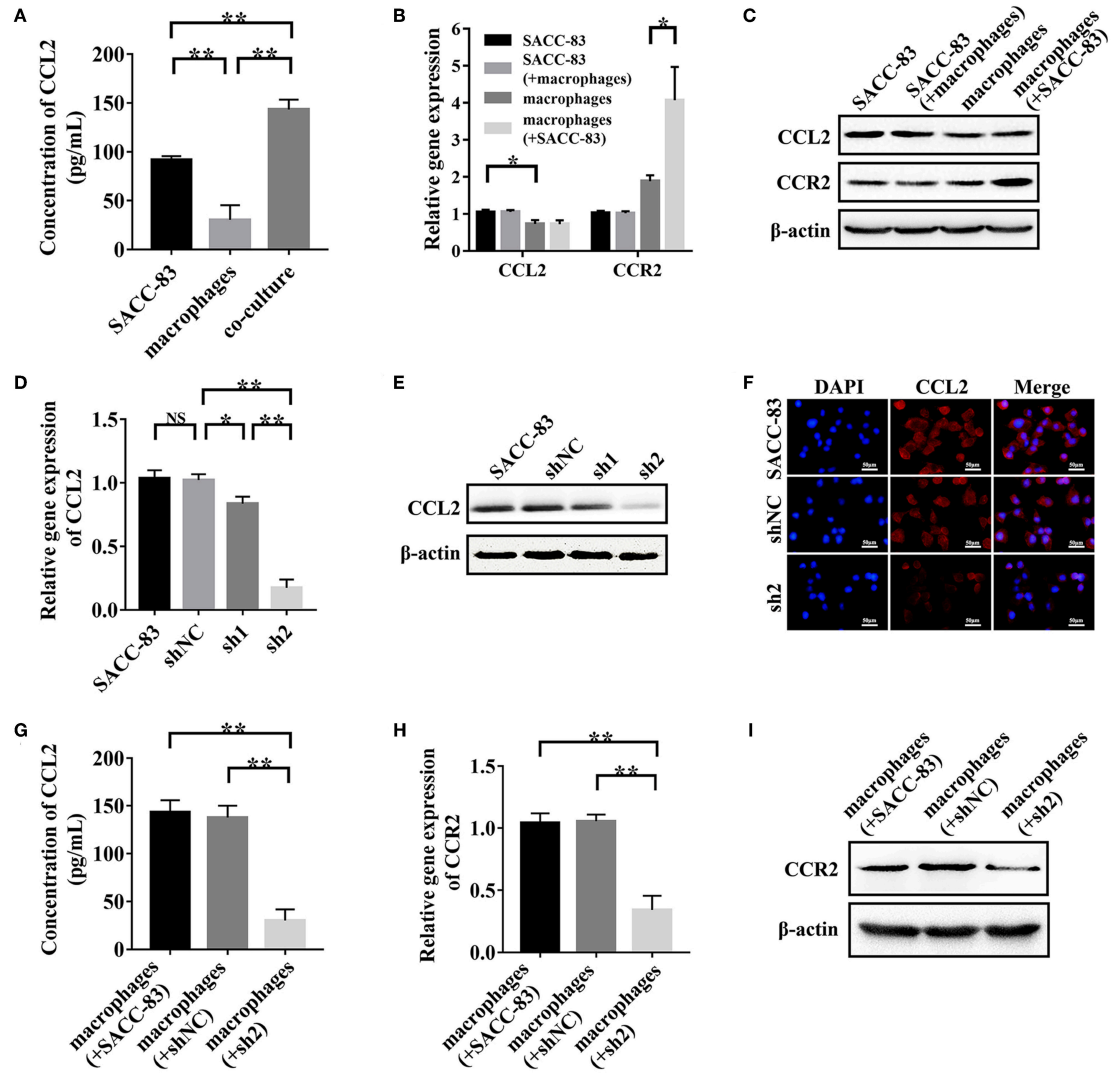
SACC cells derived CCL2 activated the expression of CCR2 in TAMs. SACC-83 cells and macrophages were co-cultured to simulate the interactions between SACC cells and TAMs. **(A)** The concentration of CCL2 in the conditioned media was examined by ELISA. The expression of CCL2 and CCR2 in the solely or co-cultured SACC-83 cells and macrophages were examined by qRT-PCR **(B)** and western blot **(C)**. The knockdown effect of CCL2 in SACC-83 cells was measured by qRT-PCR **(D)**, western blot **(E)**, and immunofluorescence **(F)**. The concentration of CCL2 in the co-culture system was detected by ELISA **(G)**. The expression of CCR2 in the solely or co-cultured macrophages were measured by qRT-PCR **(H)** and western blot **(I)**. ^*^P < 0.05, ^**^P < 0.01, NS, no significance.

To simulate the interactions between SACC cells and TAMs, SACC-83 cells or SACC-LM cells were co-cultured with macrophages using the transwell co-culture system. ELISA results showed that the concentration of CCL2 in the co-culture system was significantly higher than that in the conditioned media of SACC-83 cells or macrophages (*P* < 0.01) (Figure 3A). The results of qRT-PCR (Figure 3B) and western blot (Figure 3C) showed that the expression of CCR2 was obviously up-regulated in the co-cultured macrophages than that in the solely cultured macrophages (*P* < 0.05).

The CCL2 expression was significantly higher in SACC-83 cells than that in the macrophages (*P* < 0.05), as evaluated by ELISA (Figure 3A), qRT-PCR (Figure 3B), and western blot (Figure 3C). The qRT-PCR (Figure 3D), western blot (Figure 3E), and immunofluorescence (Figure 3F) results showed that the transduction of shRNA1 and shRNA2 significantly inhibited the CCL2 expression in sh1 cells and sh2 cells (*P* < 0.05). Whereas, the negative shRNA exhibited no interference effect in shNC cells (*P* > 0.05). The expression of CCL2 reduced more strikingly in sh2 cells than that in sh1 cells (*P* < 0.01).

The results of ELISA (Figure 3G) showed that the concentration of CCL2 was significantly higher in the conditioned media of the macrophages+SACC-83 group than that of the macrophages+sh2 group (*P* < 0.01). Whereas, the CCL2 concentration in the conditioned media has no significant difference between the macrophages+SACC-83 group and the macrophages+shNC group (*P* > 0.05). The results of qRT-PCR (Figure 3H) and western blot (Figure 3I) showed that the CCR2 expression was significantly increased in the macrophages when co-cultured with SACC-83 cells or shNC cells (*P* < 0.01), but not with sh2 cells (*P* > 0.05).

SACC-LM cells were used to validate the above findings in SACC-83 cells, and the results were in accordance with these of SACC-83 cells (Figure S1).

### CCL2/CCR2 Axis Modulated the Recruitment, M2 Polarization, and GDNF Expression of TAMs

**Figure 4:**
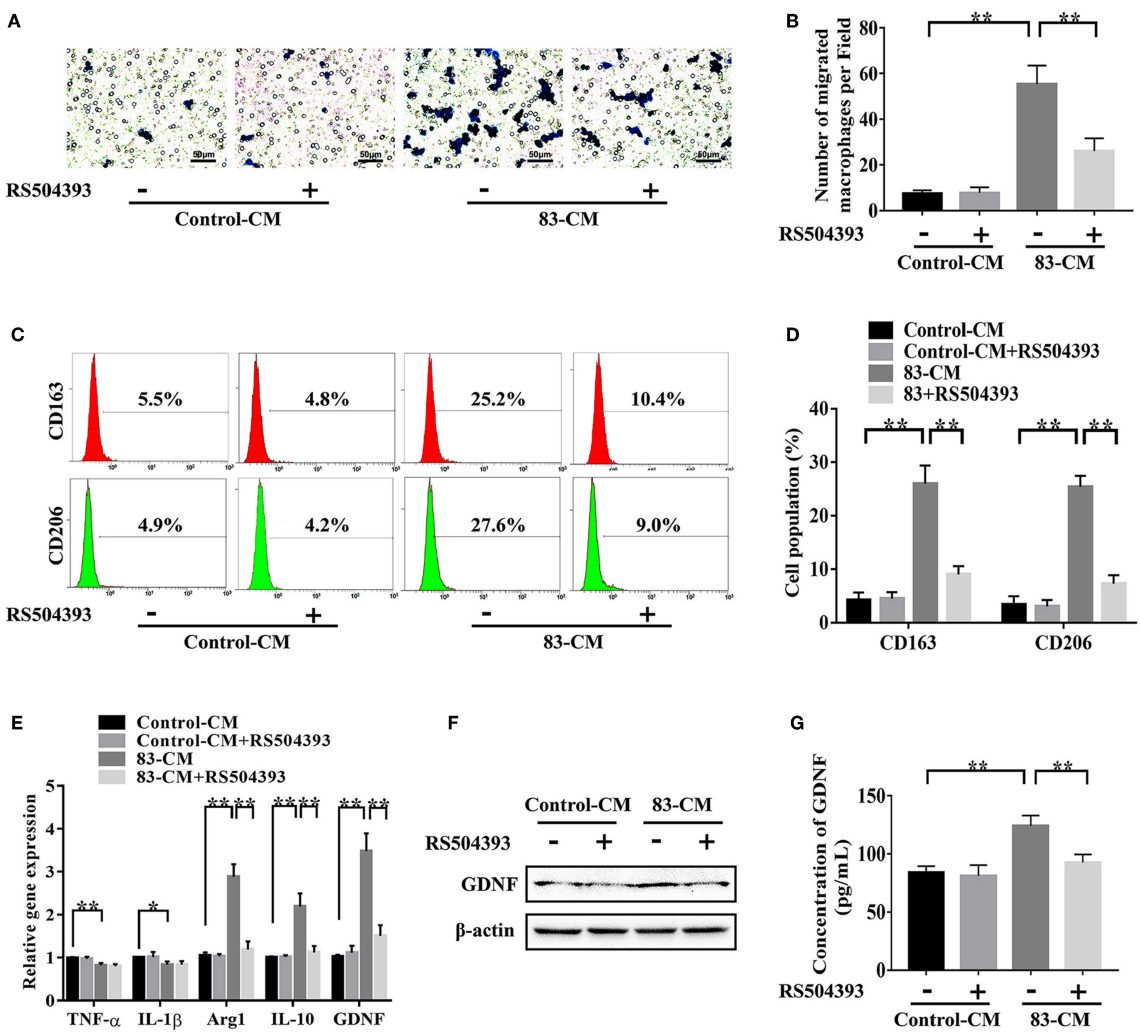
CCL2/CCR2 axis modulated the recruitment, M2 polarization, and GDNF expression of TAMs. To investigate the effect of tumor-derived CCL2 on the biological functions of TAMs, macrophages were incubated with the SACC-83 conditioned media (83-CM) with/without the treatment of CCR2 antagonist RS504393 (50 ng/mL). The serum-free 1640 media containing 0.1% BSA served as the control. **(A,B)** The recruitment of macrophages by the 83-CM was evaluated by transwell migration assays. Bar = 50 μm. **(C,D)** The percentage of CD163^+^ macrophages and CD206^+^ macrophages were evaluated by flow cytometry analysis. **(E)** The expression of TNF-α, IL-1β, Arg1, IL-10, and GDNF in macrophages were measured by qRT-CR. The expression of GDNF in macrophages was further evaluated by western blot **(F)** and ELISA **(G)**. ^*^P < 0.05, ^**^P < 0.01.

To explore the effect of tumor-derived CCL2 on the biological functions of TAMs, macrophages were incubated with the SACC-83 conditioned media or with the control media. As shown in Figures 4A,B, the SACC-83 conditioned media recruited more macrophages than the control media (*P* < 0.01). Whereas, the CCR2 antagonist RS504393 (50 ng/mL) significantly inhibited the recruitment of macrophages (*P* < 0.01).

As shown in Figures 4C,D, the flow cytometry analysis showed that the SACC-83 conditioned media significantly increased the percentage of M2 macrophages labeled by CD163 and CD206 (*P* < 0.01). Whereas, the CCR2 antagonist RS504393 (50 ng/mL) significantly reduced the percentage of M2 macrophages (*P* < 0.01). The qRT-PCR results (Figure 4E) showed that the SACC-83 conditioned media up-regulated the expression of Arg1 and IL-10 [markers for M2 polarization (24, 25)] in macrophages (*P* < 0.01), while down-regulated the expression of TNF-α and IL-1β [markers for M1 polarization (26, 27)] (*P* < 0.05). Whereas, the CCR2 antagonist RS504393 (50 ng/mL) significantly reduced the expression of Arg1 and IL-10 in macrophages (*P* < 0.01).

As shown in Figures 4E–G, the SACC-83 conditioned media significantly promoted the expression of GDNF in macrophages (*P* < 0.01), whereas the CCR2 antagonist RS504393 (50 ng/mL) significantly reduced the expression of GDNF in macrophages (*P* < 0.01).

### TAMs Promoted the Proliferation, Migration, and Invasion of SACC Cells via the GDNF/p-RET Pathway

**Figure 5:**
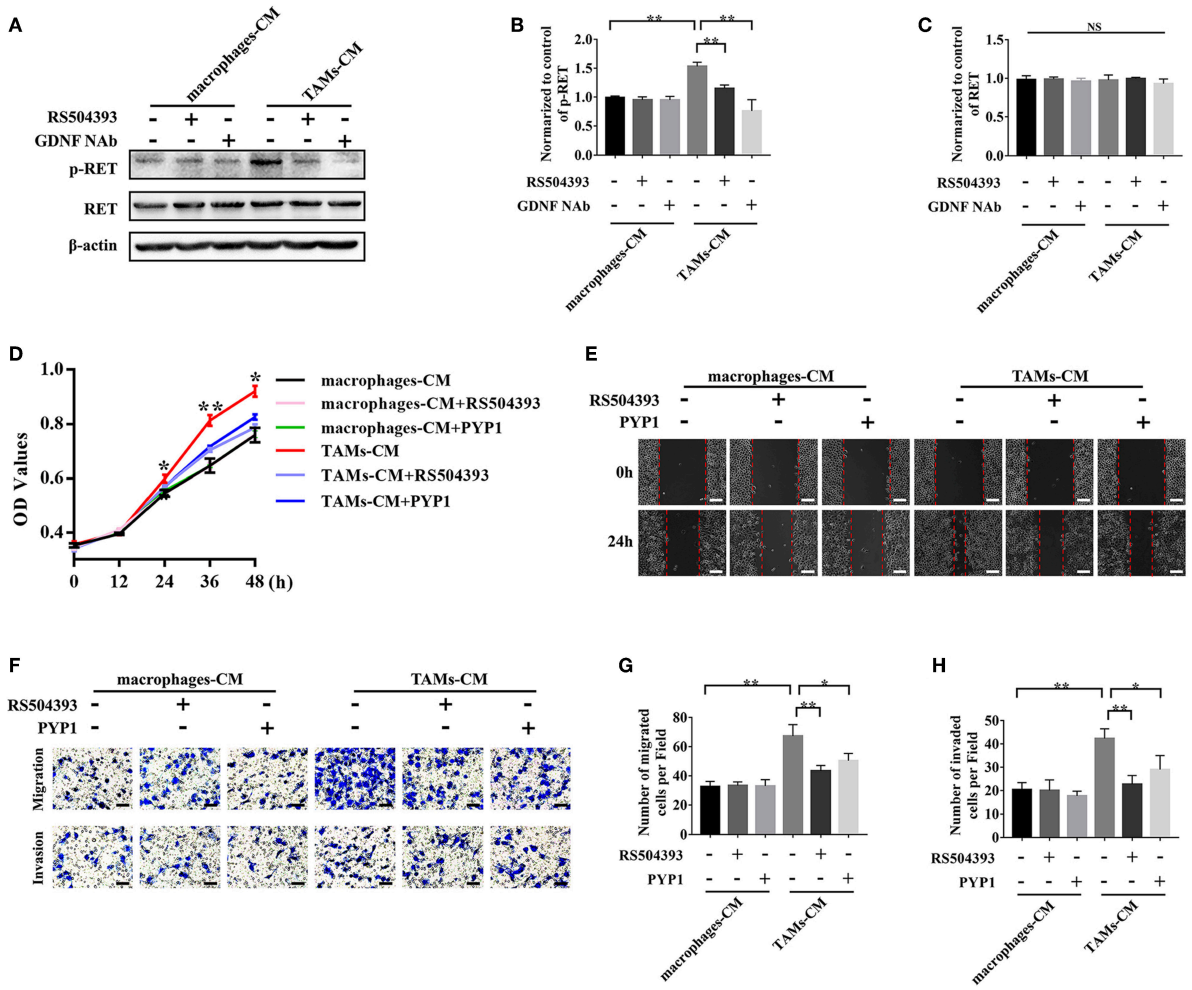
TAMs promoted the proliferation, migration, and invasion of SACC cells via the GDNF/p-RET pathway. The TAMs conditioned media (TAMs-CM) was used to stimulate the SACC-83 cells. The macrophages conditioned media (macrophages-CM) served as the control. The CCR2 antagonist (RS504393, 50 ng/mL), GDNF NAb (2 μg/mL), and RET antagonist (PYP-1, 5 μM) were used. **(A–C)** The expression of p-RET and RET in SACC-83 cells were evaluated by western blot. **(D)** The proliferation of SACC-83 cells was measured by CCK8 assays. **(E)** The motility of SACC-83 cells was evaluated by scratch wound healing assays. **(F–H)** The migration and invasion abilities of SACC-83 cells were evaluated by the transwell system. Bar = 50 μm. ^*^P < 0.05, ^**^P < 0.01, NS, no significance.

To investigate whether the TAMs derived GDNF affected the biological functions of SACC cells, SACC-83 cells were incubated with the TAMs conditioned media or with the control macrophages conditioned media. As shown in Figures 5A–C, the TAMs conditioned media significantly increased the expression of p-RET in SACC-83 cells compared with the control (*P* < 0.01). Whereas, the treatment of GDNF neutralizing antibody (GDNF NAb, 2 μg/mL) in the TAMs conditioned media or the treatment of macrophages with the CCR2 antagonist RS504393 (50 ng/mL) significantly inhibited the expression of p-RET in SACC-83 cells (*P* < 0.01).

The subsequent CCK8 (Figure 5D), scratch wound healing (Figure 5E), migration (Figures 5F,G), and invasion (Figures 5F,H) analysis showed that the TAMs conditioned media obviously promoted the proliferation, migration, and invasion of SACC-83 cells (*P* < 0.01). Whereas, the treatment of SACC-83 cells with the RET inhibitor PYP1 (5 μM) or the treatment of macrophages with the CCR2 antagonist RS504393 (50 ng/mL) obviously inhibited the proliferation, migration, and invasion of SACC-83 cells (*P* < 0.05).

### Application of the CCR2 Antagonist Attenuated the Infiltration of TAMs and the Tumorigenicity of SACC Cells

**Figure 6:**
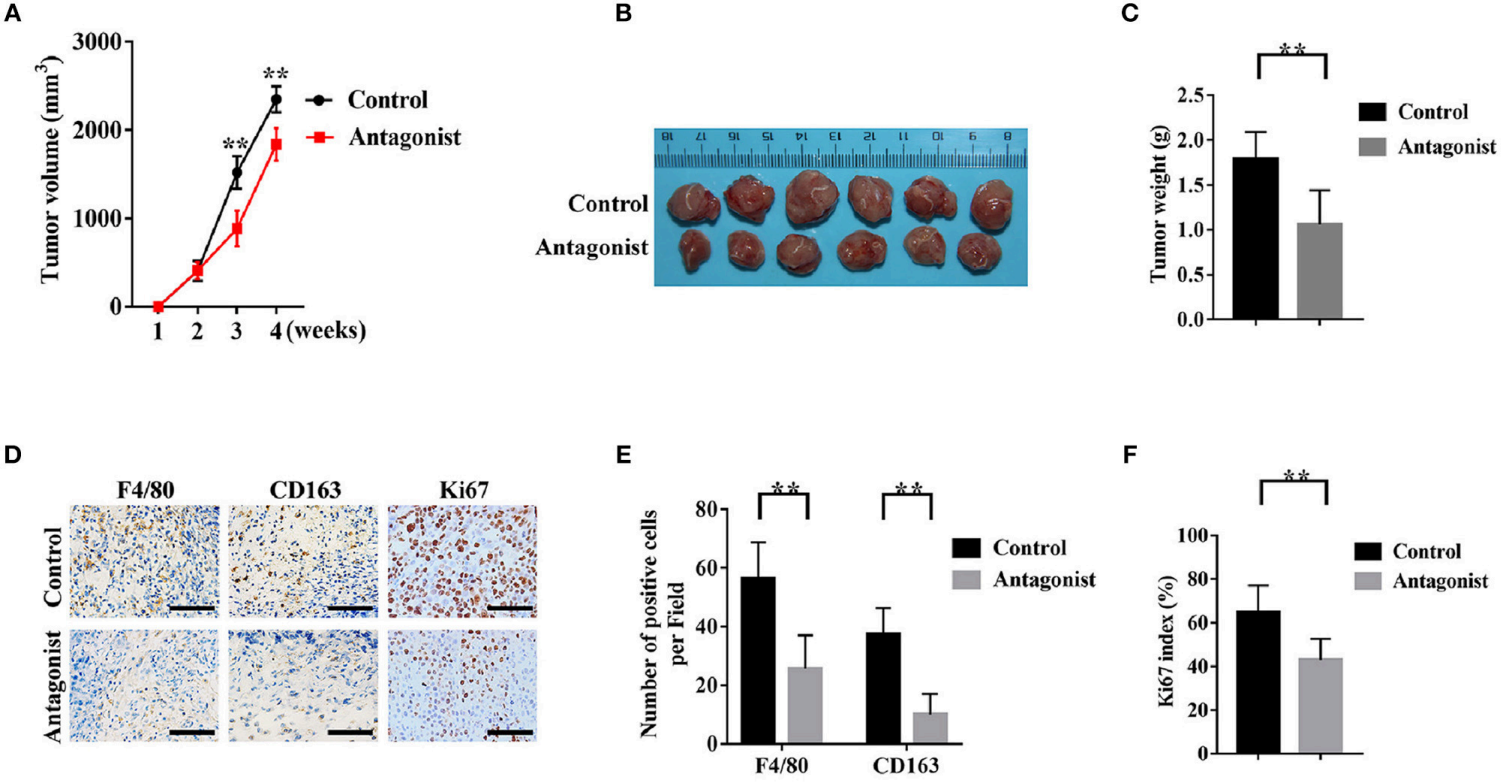
The CCR2 antagonist attenuated the infiltration of TAMs and the tumorigenicity of SACC cells. A number of 5 × 10^6^ SACC-83 cells were injected subcutaneously in the back of the male nude mice (*n* = 6 per group). One week after the inoculation, the mice were administrated orally with RS504393 (30 mg/kg) every 3rd day for 3 weeks. Mice in the control group were administrated with the corresponding solvent. **(A)** Analysis of the weekly tumor volumes. **(B)** Gross observation of the harvested tumor tissues after 4 weeks. **(C)** Analysis of the weight of the harvested tumor specimens. **(D–F)** Immunohistochemical analysis of F4/80, CD163, and Ki-67 in the harvested tumor tissues. Bar = 50μm. ^**^P < 0.01.

We established the tumor xenograft mice model with SACC-83 cells to investigate whether blocking the CCL2/CCR2 axis by the CCR2 antagonist could inhibit the development of SACC. As shown in Figures 6A–C, the CCR2 antagonist RS504393 significantly reduced the tumor volume and weight compared with the control group (*P* < 0.05). Meanwhile, the number of F4/80 labeled TAMs (Figures 6D,E), the number of CD163 labeled M2 TAMs (Figures 6D,E), and the Ki-67 index (Figures 6D,F) in the tumor tissues were significantly reduced in the CCR2 antagonist group than those in the control group (*P* < 0.01).

## Discussion

SACC is one of the most notorious salivary gland malignancies with pessimistic prognosis (28–30). Despite a series of related factors identified, the mechanism governing the aggressive behavior of SACC remains largely unknown (5, 29, 31). It is increasingly appreciated that the tumor progression is a complex process of dynamic interactions between tumor cells and the microenvironment (32–34). In addition, Substantial evidences demonstrated that TAMs are among the most important players in the tumor microenvironment (35–37). Notably, TAMs-related therapies were considered to be promising strategies for the treatment of malignant tumors (38–40). The present study for the first time demonstrated the clinical value of TAMs in the progression and prognosis of SACC.

Accumulated documents indicated that the CCL2/CCR2 axis is an important mediator in the interactions between tumor cells and TAMs (41, 42). The present study found high infiltration of TAMs in SACC by immunohistochemistry and transmission electron microscopy. The immunohistochemical analysis indicated that both CCL2 and CCR2 were overexpressed in SACC tissues. Notably, we found that the high infiltration of TAMs marked by CD68 and high infiltration of M2 TAMs marked by CD163 were correlated with the overexpression of CCL2 and CCR2. Additionally, the overexpression of CCL2, CD68, and CD163 were associated with the clinical progression and the poor prognosis of SACC patients. These data suggested that the CCL2/CCR2 axis might play critical roles in the interactions between SACC and TAMs during the progression of SACC.

CCL2 was reported to be a potent chemotactic factor for TAMs (41, 43). Recent studies found that the CCL2/CCR2 axis also regulated the M2 polarization of TAMs (16, 17, 44). The M2 polarization of TAMs was demonstrated to be associated with the aggressive behavior of malignant tumors (45, 46). In the present study, CD68 was used as the pan-macrophage marker and CD163 was used as the M2 macrophage marker. The immunohistochemical results indicated that the expression of CCL2 and CCR2 were both correlated with the number of infiltrated TAMs marked by CD68 or the number of infiltrated M2 TAMs marked by CD163 in SACC tissues. Co-culture of TAMs with SACC cells with/without CCL2 silencing indicated that SACC cells derived CCL2 could activate the CCR2 expression in TAMs. Subsequent study found that TAMs could be recruited by the SACC conditioned media, which contained high level of CCL2. While blocking the CCL2/CCR2 axis by the specific CCR2 antagonist obviously reduced the recruitment and the M2 polarization of TAMs. These results indicated that SACC cells could recruit and educate TAMs via the CCL2/CCR2 axis, which contributed to the construction of the tumor-promoting environment.

**Figure 7:**
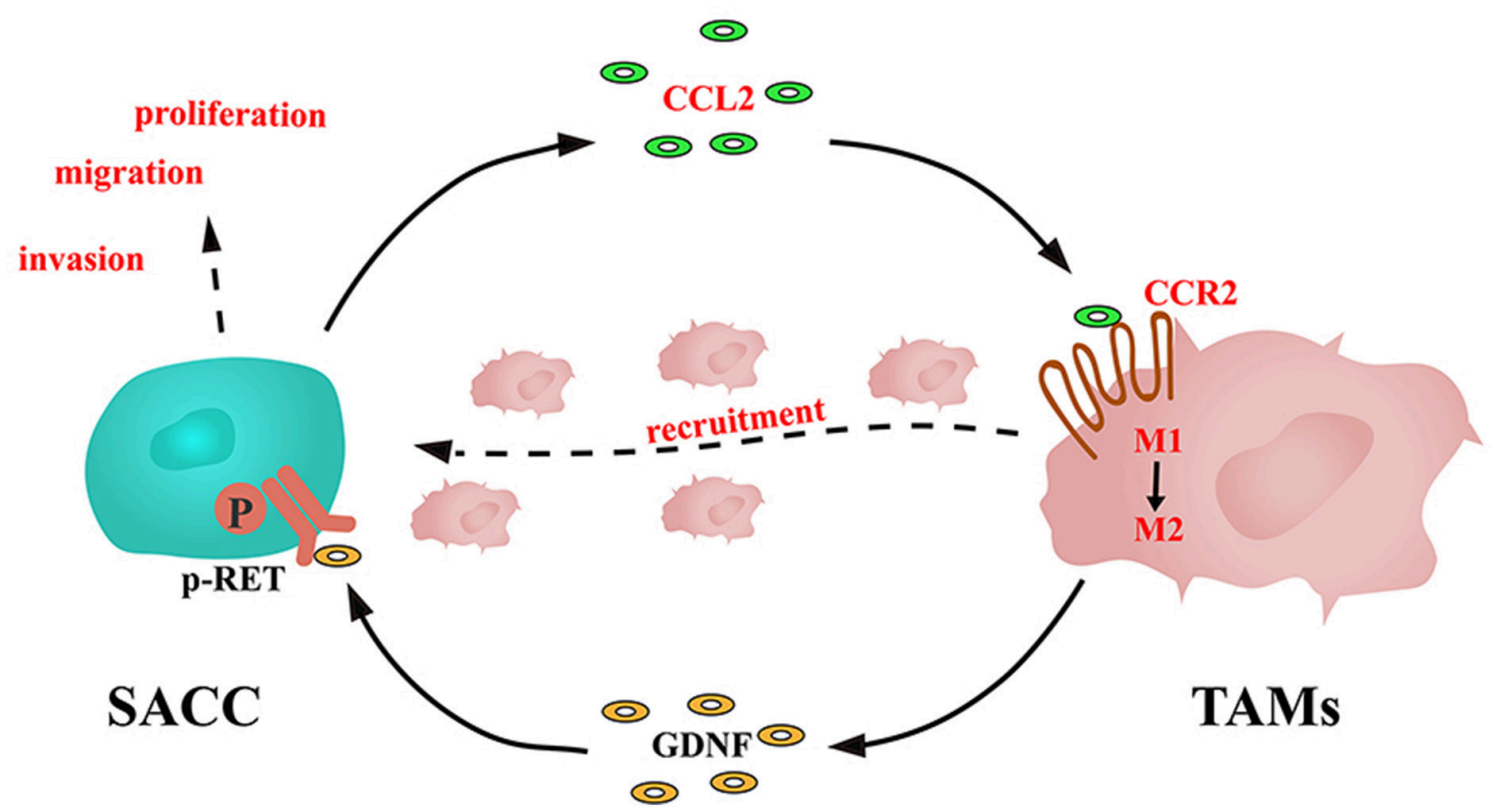
Schematic illustration of the interactions between SACC cells and TAMs mediated by CCL2/CCR2 axis.

TAMs were documented to express a high level of GDNF, which could promote the proliferation and invasion of the tumor cells (22, 47). In the present study, we noticed that the SACC conditioned media promoted the GDNF expression in TAMs. Whereas, blockade of the CCL2/CCR2 axis by the CCR2 antagonist significantly inhibited the expression of GDNF in TAMs. GDNF was demonstrated to promote the proliferation and invasion of tumor cells through phosphorylating its receptor RET (48). We found that the TAMs conditioned media promoted the phosphorylation of RET in SACC cells, which could be blocked by the GDNF NAb or the CCR2 antagonist. In addition, the CCR2 antagonist or the RET antagonist significantly inhibited the proliferation, migration, and invasion of SACC cells. Based on these results, a novel loop was identified in the interactions between SACC cells and TAMs, as shown in Figure 7: SACC cells derived CCL2 activated the expression of CCR2 in TAMs, then promoted the recruitment and M2 polarization of TAMs. Meanwhile, TAMs derived GDNF stimulated the p-RET expression in SACC cells, then promoted the aggressive behavior of SACC cells.

TAMs are supposed to be promising targets for curbing the progression of the malignant tumors (49, 50). Blockade of the CCL2/CCR2 axis has exhibited efficacy in TAMs-related therapy with several preclinical tumor models (11, 15, 51). A recent study found that application of the CCR2 antagonist inhibited the tumorigenicity via reducing the infiltration of TAMs and increasing the infiltration of cytotoxic CD8 positive lymphocytes in a murine hepatocellular carcinoma model (11). Moreover, a phase 1b trial found that the CCR2 antagonist in combination with the FOLFIRINOX chemotherapy was safe and tolerable in the treatment of pancreatic cancer, which resulted in an endogenous anti-tumor environment (18). In the present study, we found that blocking the CCL2/CCR2 axis by the CCR2 antagonist reduced the infiltration of TAMs and inhibited the tumorigenicity of SACC cells. These results suggested that targeting the CCL2/CCR2 axis might be a potential therapeutic strategy for SACC patients. Further investigations are still needed to get more details about the interactions between SACC and TAMs to achieve safe and efficient TAMs-related therapy for SACC.

In conclusion, the present study demonstrated that the infiltration of TAMs markedly correlated with the progression and the poor prognosis of SACC. Our findings suggested that the CCL2/CCR2 axis promoted the progression of SACC via recruiting and reprogramming TAMs. Targeting TAMs by blocking the CCL2/CCR2 axis might be a potential therapeutic option for SACC.

## Ethics Statement

All procedures involving animals have been examined and certified by the Committee on Use of Live Animals for Teaching and Research of the Fourth Military Medical University (Ethics Reference NO: 2017-011). All procedures involving human participants were approved by the Medical Research Ethics Committee of The Fourth Military Medical University (Ethics Reference NO: IRB-REV-2017016). The written informed consent was obtained from all participants included in the study, in agreement with institutional guidelines.

## Author Contributions

ZY was responsible for designing and conducting all experiments, interpreting the data, and writing the manuscript. HL and WW assisted the *in vitro* studies and contributed to the data acquisition and analysis. JZ and SJ assisted both *in vitro* and *in vivo* studies. JuW, JiW, and DL collected the clinical data. XY and KH assisted experimental design and were responsible for research supervision. All authors read and approved the final manuscript.

### Conflict of Interest Statement

The authors declare that the research was conducted in the absence of any commercial or financial relationships that could be construed as a potential conflict of interest.

## Supplementary Material

Figure S1 SACC-LM cells derived CCL2 activated the expression of CCR2 in TAMs. SACC-LM cells and macrophages were co-cultured to simulate the interactions between SACC-LM cells and TAMs. **(A)** The concentration of CCL2 in the conditioned media was examined by ELISA. The expression of CCL2 and CCR2 in the solely or co-cultured SACC-LM cells and macrophages were examined by qRT-PCR **(B)** and western blot **(C)**. The knockdown effect of CCL2 in SACC- LM cells was measured by qRT-PCR **(D)**, western blot **(E)**, and immunofluorescence **(F)**. The concentration of CCL2 in the co-culture system was detected by ELISA **(G)**. The expression of CCR2 in the solely or co-cultured macrophages were measured by qRT-PCR **(H)** and western blot **(I)**. ^*^*P* < 0.05, ^**^*P* < 0.01, NS, no significance.

Table S1 Sequences of CCL2-specific shRNAs.

Table S2 Primer used for Real-time PCR analysis.
