# Supplementary figures and images for: CCL2/CCR2 Axis Promotes the Progression of Salivary Adenoid Cystic Carcinoma via Recruiting and Reprogramming the Tumor-Associated Macrophages
Source: Front Oncol. 2019 Apr 9;9:231. doi: 10.3389/fonc.2019.00231 (PMC6465613; doi:10.3389/fonc.2019.00231)

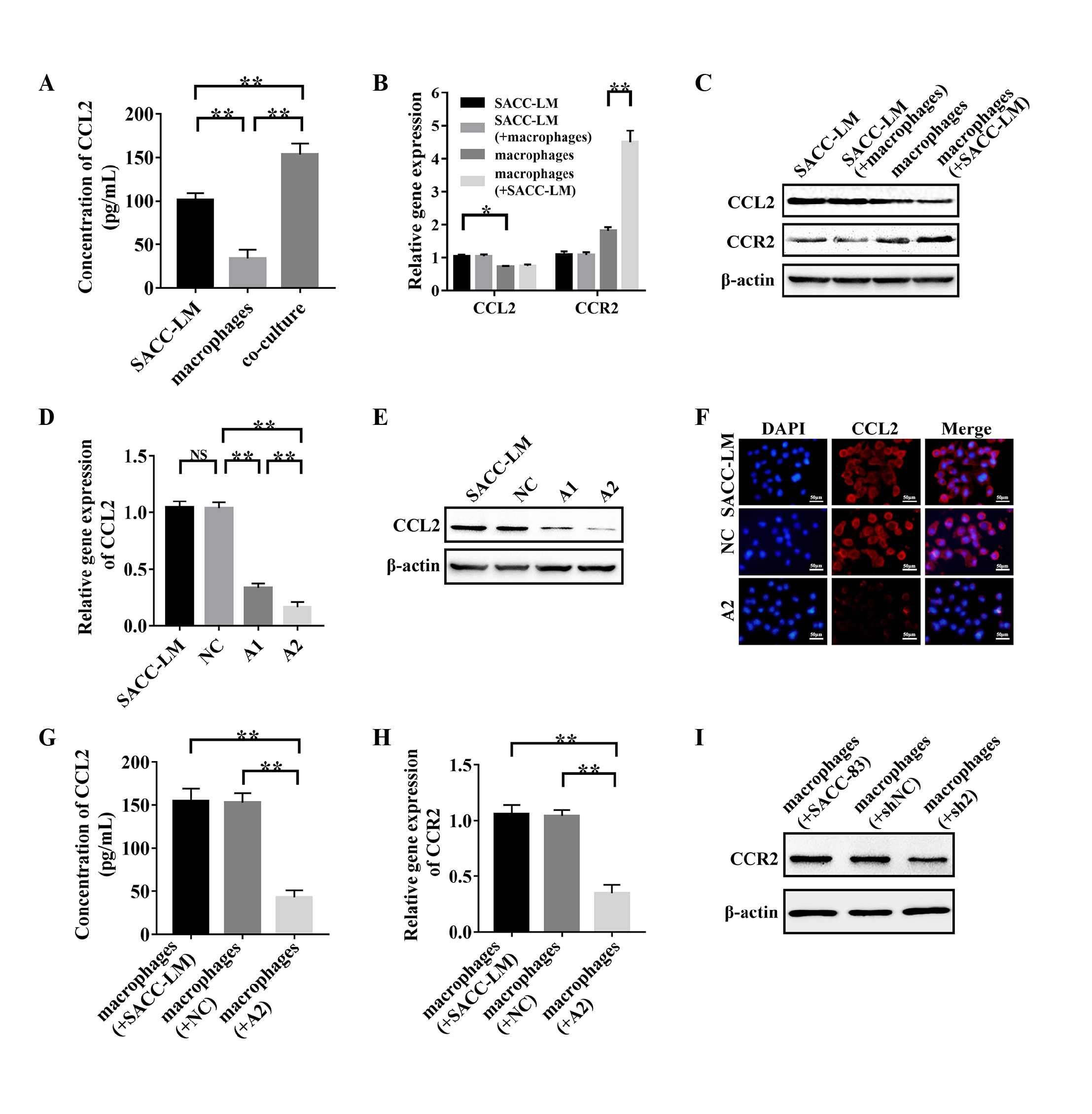

Supplement: Figure S1 — SACC-LM cells derived CCL2 activated the expression of CCR2 in TAMs. SACC-LM cells and macrophages were co-cultured to simulate the interactions between SACC-LM cells and TAMs. (A) The concentration of CCL2 in the conditioned media was examined by ELISA. The expression of CCL2 and CCR2 in the solely or co-cultured SACC-LM cells and macrophages were examined by qRT-PCR (B) and western blot (C). The knockdown effect of CCL2 in SACC- LM cells was measured by qRT-PCR (D), western blot (E), and immunofluorescence (F). The concentration of CCL2 in the co-culture system was detected by ELISA (G). The expression of CCR2 in the solely or co-cultured macrophages were measured by qRT-PCR (H) and western blot (I). *P < 0.05, **P < 0.01, NS, no significance. [file Image_1.JPEG]
