# Supplementary material for: CCL2/CCR2 Axis Promotes the Progression of Salivary Adenoid Cystic Carcinoma via Recruiting and Reprogramming the Tumor-Associated Macrophages
Source: Front Oncol. 2019 Apr 9;9:231. doi: 10.3389/fonc.2019.00231 (PMC6465613; doi:10.3389/fonc.2019.00231)
Supplement: Table S1 — Sequences of CCL2-specific shRNAs. [file Table_1.DOCX]

­­­**TABLE S1** Sequences of CCL2-specific shRNAs.

| **Name** | **Sense/antisense Sequences of shRNA** | **Target necleotides site** |
| --- | --- | --- |
| CCL2-shNC | Forward 5’-CCGGAACAGTCGCGTTTGCGACTGGCTCGAGC CAGTCGCAAACGCGACTGTTTTTTTG-3’  Reverse 5’-AATTCAAAAAAACAGTCGCGTTTGCGACTGGCT CGAGCCAGTCGCAAACGCGACTGTT-3’ | None |
| CCL2-shRNA1 | Forward 5’-CCGGTCATAGCAGCCACCTTCATTCCTCGAGGA ATGAAGGTGGCTGCTATGATTTTTG-3’  Reverse 5’-AATTCAAAAATCATAGCAGCCACCTTCATTCCTC GAGGAATGAAGGTGGCTGCTATGA-3’ | 35-55 |
| CCL2-shRNA2 | Forward 5’-CCGGCCCAGTCACCTGCTGTTATAACTCGAGTT ATAACAGCAGGTGACTGGGTTTTTG-3’  Reverse 5’-AATTCAAAAACCCAGTCACCTGCTGTTATAACTC GAGTTATAACAGCAGGTGACTGGG-3’ | 89-110 |
