# Supplementary material for: CCL2/CCR2 Axis Promotes the Progression of Salivary Adenoid Cystic Carcinoma via Recruiting and Reprogramming the Tumor-Associated Macrophages
Source: Front Oncol. 2019 Apr 9;9:231. doi: 10.3389/fonc.2019.00231 (PMC6465613; doi:10.3389/fonc.2019.00231)
Supplement: Table S2 — Primer used for Real-time PCR analysis. [file Table_2.DOCX]

**TABLE S2** Primer used for Real-time PCR analysis.

| **mRNA** | **size** | **Primer sequence** |
| --- | --- | --- |
| CCL2 | 115 | Forward 5’- GAGGAACCGAGAGGCTGAGA -3’ |
|  |  | Reverse 5’- GCTATGAGCAGCAGGCACAG -3’ |
| CCR2 | 152 | Forward 5’- TTAGTTGCCCTGTATCTCCGC-3’  Reverse 5’- CTCTGTTCAGCTTGTGGCTTG-3’ |
| TNF-α | 144 | Forward 5’- GCTGCACTTTGGAGTGATCG-3’ |
|  |  | Reverse 5’- GCTTGAGGGTTTGCTACAACA-3’ |
| IL-1β | 104 | Forward 5’- AAGTACCTGAGCTCGCCAGT-3’ |
|  |  | Reverse 5’-CTGGAAGGAGCACTTCATCTGT-3’ |
| Arg1 | 121 | Forward 5’- AGGAATTGGCAAGGTGATGG-3’ |
|  |  | Reverse 5’- TGTGCCAGTAGCTGGTGTGA-3’ |
| IL-10 | 130 | Forward 5’- GCCTGGTCCTCCTGACTG-3’ |
|  |  | Reverse 5’- TTCACTCTGCTGAAGGCATC-3’ |
| GDNF | 115 | Forward 5’-CCTAGAAGAGAGCGGAATCG-3’ |
|  |  | Reverse 5’- AATGTATTGCAGTTAAGACACAACC-3’ |
| β-actin | 205 | Forward 5’- TGACGTGGACATCCGCAAAG-3’ |
|  |  | Reverse 5’- CTGGAAGGTGGACAGCGAGG-3’ |
